# Exploration of resting‐state brain functional connectivity as preclinical markers for arousal prediction in prolonged disorders of consciousness: A pilot study based on functional near‐infrared spectroscopy

**DOI:** 10.1002/brb3.70002

**Published:** 2024-08-25

**Authors:** Yaomin Luo, Lingling Wang, Yuxuan Yang, Xin Jiang, Kaiyuan Zheng, Yu Xi, Min Wang, Li Wang, Yanlin Xu, Jun Li, Yulei Xie, Yinxu Wang

**Affiliations:** ^1^ Department of Rehabilitation Medicine Affiliated Hospital of North Sichuan Medical College Nanchong China; ^2^ Department of Rehabilitation Medicine West China Second Hospital of Sichuan University Chendu China; ^3^ Department of Respiratory Medicine Gaoping District People's Hospital Nanchong China; ^4^ Department of Operating Room Nanchong Hospital of Traditional Chinese Medicine Nanchong China; ^5^ Department of Paediatric Surgery Nanchong Central Hospital, The Second Clinical College, North Sichuan Medical College Nanchong China; ^6^ Sports Rehabilitation, North Sichuan Medical College Nanchong China; ^7^ School of Rehabilitation Capital Medical University Beijing China

**Keywords:** brain networks, functional near‐infrared spectroscopy, prolonged disorders of consciousness, resting state functional connectivity

## Abstract

**Background:**

There is no diagnostic assessment procedure with moderate or strong evidence of use, and evidence for current means of treating prolonged disorders of consciousness (pDOC) is sparse. This may be related to the fact that the mechanisms of pDOC have not been studied deeply enough and are not clear enough. Therefore, the aim of this study was to explore the mechanism of pDOC using functional near‐infrared spectroscopy (fNIRS) to provide a basis for the treatment of pDOC, as well as to explore preclinical markers for determining the arousal of pDOC patients.

**Methods:**

Five minutes resting‐state data were collected from 10 pDOC patients and 13healthy adults using fNIRS. Based on the concentrations of oxyhemoglobin (HbO) and deoxyhemoglobin (HbR) in the time series, the resting‐state cortical brain functional connectivity strengths of the two groups were calculated, and the functional connectivity strengths of homologous and heterologous brain networks were compared at the sensorimotor network (SEN), dorsal attention network (DAN), ventral attention network (VAN), default mode network (DMN), frontoparietal network (FPN), and visual network (VIS) levels. Univariate binary logistic regression analyses were performed on brain networks with statistically significant differences to identify brain networks associated with arousal in pDOC patients. The receiver operating characteristic (ROC) curves were further analyzed to determine the cut‐off value of the relevant brain networks to provide clinical biomarkers for the prediction of arousal in pDOC patients.

**Results:**

The results showed that the functional connectivity strengths of oxyhemoglobin (HbO)‐based SEN∼SEN, VIS∼VIS, DAN∼DAN, DMN∼DMN, SEN∼VIS, SEN∼FPN, SEN∼DAN, SEN∼DMN, VIS∼FPN, VIS∼DAN, VIS∼DMN, HbR‐based SEN∼SEN, and SEN∼DAN were significantly reduced in the pDOC group and were factors that could reflect the participants' state of consciousness. The cut‐off value of resting‐state functional connectivity strength calculated by ROC curve analysis can be used as a potential preclinical marker for predicting the arousal state of subjects.

**Conclusion:**

Resting‐state functional connectivity strength of cortical networks is significantly reduced in pDOC patients. The cut‐off values of resting‐state functional connectivity strength are potential preclinical markers for predicting arousal in pDOC patients.

## Graphical abstract

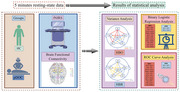

## 1 INTRODUCTION

Disorders of consciousness (DOC) are the most common complication of severe brain injury, with prolonged disorders of consciousness (pDOC) defined as DOC for more than 28 days (Song et al., 2020), including the unresponsive wakefulness syndrome (UWS) and the minimally conscious state (MCS) (Matsumoto‐Miyazaki et al., 2016). Relevant epidemiological studies have reported that ∼10%–15% of patients develop pDOC after brain injury (Andriessen et al., 2011; Y. Li et al., 2015). They may die or regain consciousness or may remain in the state of unconscious or minimally conscious for longer. Its uncertain efficacy, difficult to judge prognosis, and heavy economic burden pose many social and economic problems for clinical decision‐making (Xie, Wang, Jia et al., 2022; Xie, Wang, Xie et al., 2022). However, there is no diagnostic assessment procedure with moderate or strong evidence of use (Estraneo et al., 2017). Standardized behavior assessment still is the “gold standard” for detecting signs of consciousness (Morrissey et al., 2018). But even the most accepted and recommended assessment of Coma Recovery Scale‐Revised (CRS‐R) may lead to the misdiagnosis rate of pDOC patients reaching 36% (Wannez et al., 2017a). It is possible that a positive electromyogram response to command, electroencephalogram (EEG) reactivity to sensory stimuli, laser‐evoked potentials, and the Perturbational Complexity Index can distinguish MCS from UWS (Giacino et al., 2018), but none of them provide preclinical markers for predicting arousal in pDOC patients. In addition to this, the evidence for current means of treating pDOC is sparse, and there is no consistent treatment guideline; only several drug (Giacino, Whyte, et al., 2012; Jha et al., 2008; Krimchansky et al., 2004; Passler & Riggs, 2001; Reynolds et al., 2013; Tucker & Sandhu, 2016) and non‐drug treatments (Demirtas‐Tatlidede et al., 2012; Giacino, Fins, et al., 2012; Louise‐Bender et al., 2009; Popernack et al., 2015) are available. This may be related to the fact that the mechanisms of pDOC have not been studied deeply enough and are not clear enough. Therefore, the aim of this study was to explore the mechanism of pDOC using functional near‐infrared spectroscopy (fNIRS) to provide a basis for the treatment of pDOC, as well as to explore preclinical markers for predicting the arousal of pDOC patients.

In recent years, advanced neuroimaging techniques have strongly contributed to capture these changes in functional connections associated with pDOC and promoted a rapid progress in the deeper understanding of the disease. Using advanced neuroimaging technology to directly detect the brain functional network of pDOC patients by bypassing behavioral response can reveal some hidden consciousness and cognition in patients' brains earlier (Fernandez‐Espejo et al., 2015; Owen et al., 2006), thus providing a means for more accurate diagnosis and more accurate prognosis (Monti et al., 2010). Resting‐state functional connectivity (FC) has been widely used to investigate pDOC and other neurological and psychiatric diseases (L. Wang et al., 2022). Currently common tools for assessing resting‐state brain FC in pDOC patients include functional magnetic resonance imaging (fMRI) in relevant studies (Almdahl et al., 2021; Q. Chen et al., 2019; Hrybouski et al., 2021; Patil et al., 2021) and fNIRS, which facilitate the analysis of functional connections within and between brain networks without the need for subjects to perform a specific task. FMRI has excellent spatial resolution and can be better integrated with structural lesions (Ansado et al., 2021). Resting‐state functional magnetic resonance imaging (rs‐fMRI) has been recommended as a part of clinical multimodal evaluation of DOC and provides valuable information for brain network to detect possible subtle changes in brain activity (Norton et al., 2023; Snider & Edlow, 2020). Good agreement between fNIRS and fMRI results was found (Sasai et al., 2012). However, compared with fMRI, fNIRS is inexpensive, resistant to motion interference, resistant to electromagnetic interference, has high temporal and spatial resolution, and is easy to move (Cui et al., 2010). Therefore, fNIRS has great potential as a neuroimaging biomarker to enhance the diagnosis and disease monitoring of pDOC.

Research on FC has highlighted that the brain is intrinsically organized into distinct large‐scale connectivity networks, which facilitate human brain function by their dynamic interplay (Fox et al., 2005). Here, we focus on the major brain networks that have been identified in the past few years as being related to Consciousness. As we know, consciousness includes wakefulness and awareness (Naro et al., 2017), and awareness can be subdivided into two parts: environment (external) and self (internal) awareness (Demertzi et al., 2011). It has been identified that the frontoparietal network (FPN) is associated with external consciousness and that its metabolism is positively correlated with CRS‐R scores (Leonardi et al., 2018; Mencarelli et al., 2020; Panda et al., 2022). The ventral attention network (VAN) has a right lateralization, is primarily responsible for non‐spatial attention, and can participate in stimulus‐driven top‐down attentional selection (Deslauriers et al., 2017). The default mode network (DMN) exhibits internal activities, also referred to as the “task‐negative network” (Andrews‐Hanna, 2012; Andrews‐Hanna et al., 2014), whereas the lateral frontoparietal areas related to the network of dorsal attention (DAN) mediate task‐driven stimuli (Mallas et al., 2021). In another case, according to the regulating function, the brain networks could be classified into higher order networks [the DMN and DAN] and sensory‐related lower order networks including visual network (VIS) and sensorimotor network (SEN) (H. Li et al., 2023). Therefore, this study takes these six brain networks as the research object to explore the functional connection characteristics of pDOC cerebral cortex networks.

Some work has been done to accurately diagnose patients with DOC (Schnakers et al., 2009; Wannez et al., 2017b), establish prognostic indicators (Estraneo et al., 2014; Faugeras et al., 2018), and understand the neural centers of consciousness (Thibaut et al., 2019; Wagner et al., 2020). This work is critical because diagnosis determines treatment and is closely linked to functional prognosis, and misdiagnosis can lead to important medical decisions such as withdrawal of life‐sustaining care. However, the classification of pDOC is largely based on observable behavioral features and their inferred relationship to the level of consciousness, and diagnostic classification methods based on pathophysiological mechanisms have yet to be developed. Advanced neuroimaging techniques can complement clinical assessment by providing physiological evidence of brain activity. However, it remains an open question whether these empirical findings are directly or indirectly related to concealed consciousness, how to establish an efficient diagnostic protocol, and the limitations of diagnostic applications at the individual patient level. The studies on fNIRS and pDOC are even fewer and mostly limited to the validity studies of this test. The present study not only explored the functional connectivity features of the pDOC brain network but also revealed the brain network reflecting the subject's state of consciousness and its cut‐off value, which is expected to provide a theoretical basis for the accurate diagnosis of the state of consciousness, the prediction of the prognosis, the judgement of the efficacy of treatment, and the study of the mechanism.

## 2 MATERIALS AND METHODS

### 2.1 Participants

In this observational study, we recruited healthy volunteers and inpatients aged 18 and above. All participants provided written informed consent. This study was approved by the Medical Ethics Committee of the Affiliated Hospital of North Sichuan Medical College, with healthy adults as the control group (HC) and pDOC patients as the experimental group, and recruited strictly according to the inclusion and exclusion criteria.

We recruited 23 participants and selected subject data according to the following inclusion and exclusion criteria. Inclusion criteria for pDOC were (1) meeting the definition of pDOC, diagnosed as vegetative state/unresponsive awakening syndrome, and minimal consciousness according to the revised Coma Recovery Scale, and course of disease ≥ 28 days and (2) the pathogenic factors are trauma, stroke, or ischemia and hypoxia. Inclusion criteria for HC were (1)score of 27 or above on the mini‐mental state examination (MMSE) and (2) no significant visual or hearing impairment. Exclusion criteria for pDOC were (1) patients with unstable vital signs (respiratory or hemodynamic instability), combined with severe cardiac, hepatic, renal, or pulmonary impairment or other serious diseases, (2) use of central excitatory drugs within 24 h before the test, and (3) patients who are completely unable to co‐operate with the assessment. Exclusion criteria for HC were (1) combined with severe neurological disease or critical brain infarction; (2) combined with severe mental illness; (3) previous serious heart, lung, liver, kidney, and other important organ failure; (4) uncontrollable hypertension, arrhythmia, severe coronary artery disease, and poorly controlled diabetic; and (5) Poor compliance, unable to cooperate with this study.

### 2.2 Measurement tasks and execution methods

Basic information about all subjects was collected prior to assessment, including name, sex, age at baseline, symptoms, time of onset, site of stroke, current medical history, past medical history, family history of genetic disorders, previous treatments, imaging findings, and medication use. Three CRS‐R(Seel et al., 2010) scores for pDOC patients and three MMSE scores for healthy adults within 1 week, with the highest score obtained in the three assessments as the basis for inclusion in pDOC and HC(Kalmar & Giacino, 2005), were obtained by assessment of a professional investigator not involved in the other assessments.

#### 2.2.1 fNIRS measurement

**FIGURE 1:**
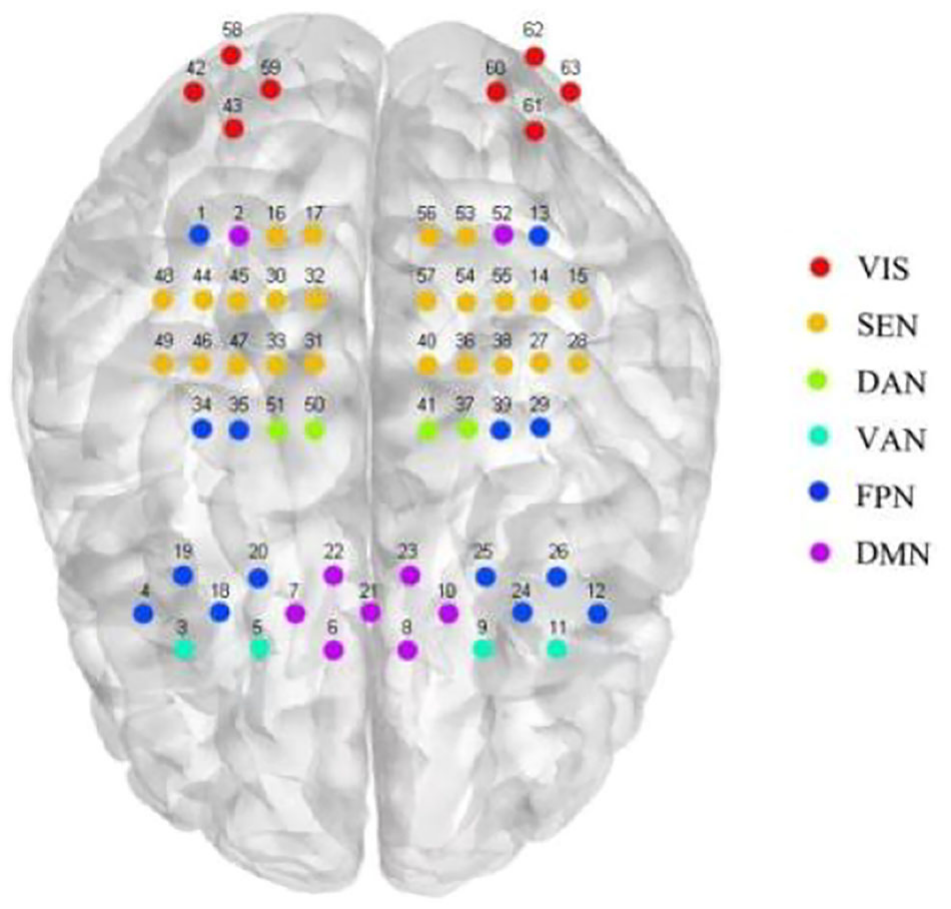
Correspondence between functional near‐infrared spectroscopy (fNIRS) acquisition headcaps and brain networks. DAN, dorsal attentional network; DMN, default mode network; FPN, frontoparietal network; SEN, sensorimotor network; VAN, ventral attentional network; VIS, visual network.

We used a 63‐channel desktop fNIRS device (Danyang Huitron) to capture the functional connectivity strength of cortical brain networks in the resting state of participants. Participants entered the assessment room and familiarized themselves with the environment for 5 min before putting on the fNIRS helmet and avoiding physical activity. The lighting in the assessment room was then switched off, and resting state data were collected from participants for 5 min (Kempny et al., 2016). The SEN, DAN, VAN, FPN, DMN, and VIS of the cerebral cortex subdivision were selected according to the coverage of fNIRS channels and consciousness‐related brain networks (Figure 1).

#### 2.2.2 Preprocessing

Preprocessing of the fNIRS resting‐state data was performed in the Preprocess module of the NirSpark software (H. Li et al., 2023; Zou et al., 2022). First, the motion artifacts were identified and removed using the spline interpolation method, and the signal standard deviation threshold was set to 6, and the peak threshold was set to 0.5. Then, the general noise including heartbeat and respiration was filtered with a band‐pass filter of 0.01–0.2 Hz. Finally, the path difference factor was set from −6 to 6, and the concentration changes of HbO and deoxyhemoglobin (HbR) in the resting state of the participant were calculated according to the modified Beer–Lamber law.

The changes of HbO and HbR concentrations in the participant at each time point of the resting state measurement were extracted in the Network module of NirSpark software, and the Pearson correlation coefficients of HbO and HbR contents of each channel on the time series were analyzed. Then, FisherZ transformation was performed, and the transformed values were defined as the functional connection strength between channels.

### 2.3 Statistical analysis

**FIGURE 2:**
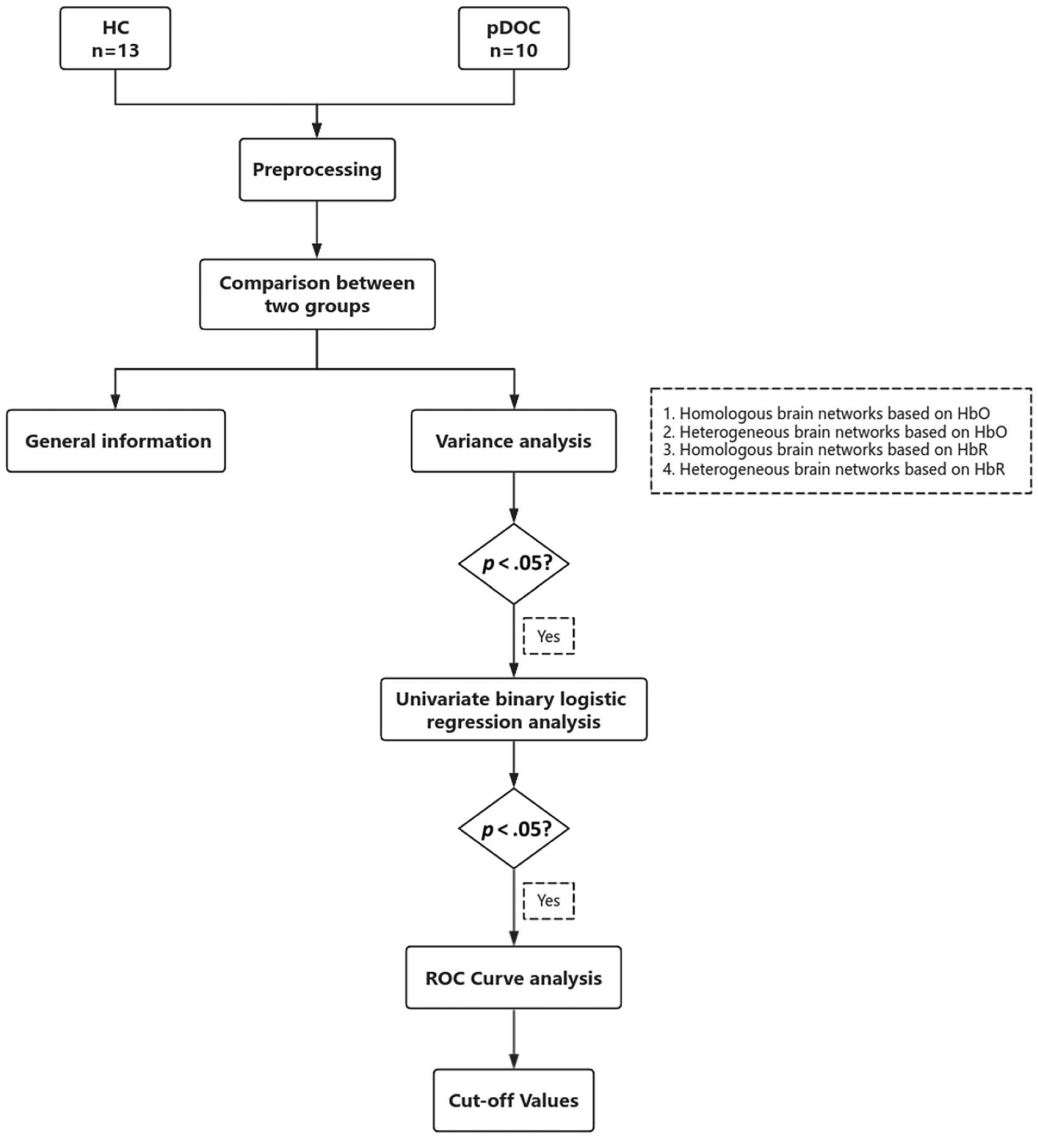
The flowchart for the data analysis. HbO, oxyhemoglobin; HbR, deoxyhemoglobin; HC, control group; pDOC, prolonged disorders of consciousness; ROC, receiver operating characteristic.

IBM SPSS Statistics for Windows (Version 27.0; IBM Corp.) was used for statistical analysis (Figure 2). Two‐tailed *p* <.05 was considered a statistically significant difference. The Shapiro–Wilk test was used to assess whether the data obeyed a normal distribution. If the measurement data obeyed normal distribution, independent samples t‐test was used for inter‐group comparison. If they did not conform to a normal distribution, the Mann–Whitney *U* test was used. Factors with statistically significant differences were then analyzed by univariate binary logistic regression, and two‐tailed *p* <.05 was considered a statistically significant difference. Finally, ROC curves were then plotted, and the area under the curve, sensitivity, and specificity were calculated, and the functional connectivity strength corresponding to the maximal Youden exponent was the optimal cut‐off value.

## 3 RESULTS

### 3.1 Comparison of general data between the two groups

Ten patients with pDOC and 13 healthy adults were finally included in this study. There was no significant difference in age and sex between the two groups (*p* >.05), and the two groups were comparable (Table 1).

**TABLE 1:** Comparison of general data between the healthy adult and prolonged disorders of consciousness (pDOC) group.

General information	HC (*n *= 13)	pDOC (*n *= 10)	*p‐*Value
Age	44.00 (27.00, 54.00)	51.50 (27.50, 64.50)	*p =*.3654
Gender			*p =*.8548
Female *n*(%)	7(53.85)	5(50)	
Male *n*(%)	6(46.15)	5(50)	

Abbreviation: HC, control group.

### 3.2 Characteristics and differences in functional connectivity of cortical brain networks between the healthy adult and pDOC groups based on HbO

**FIGURE 3:**
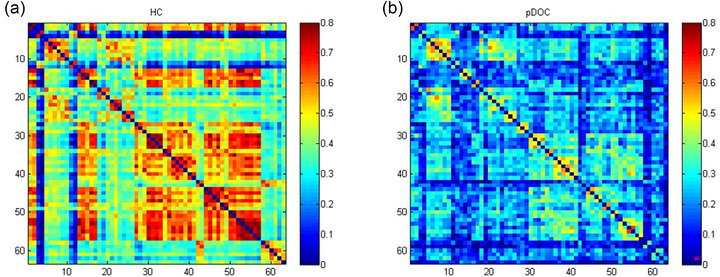
Whole‐brain mean functional connectivity strength based on oxyhemoglobin (HbO) in the healthy adult group (a) and the prolonged disorders of consciousness (pDOC) group (b).

Compared with the HC group, the strength of HbO‐based functional connectivity in the pDOC group showed a decreasing trend in all six cortical brain networks (Figures 3), and the strength of HbO‐based functional connectivity in five homologous brain networks (Figure 5a) and eight heterologous brain networks (Figure 5b) in the pDOC group was significantly lower (*p* <.05).  

### 3.3 Characteristics and differences in functional connectivity of cortical brain networks between the healthy adult and pDOC groups based on HbR

**FIGURE 4:**
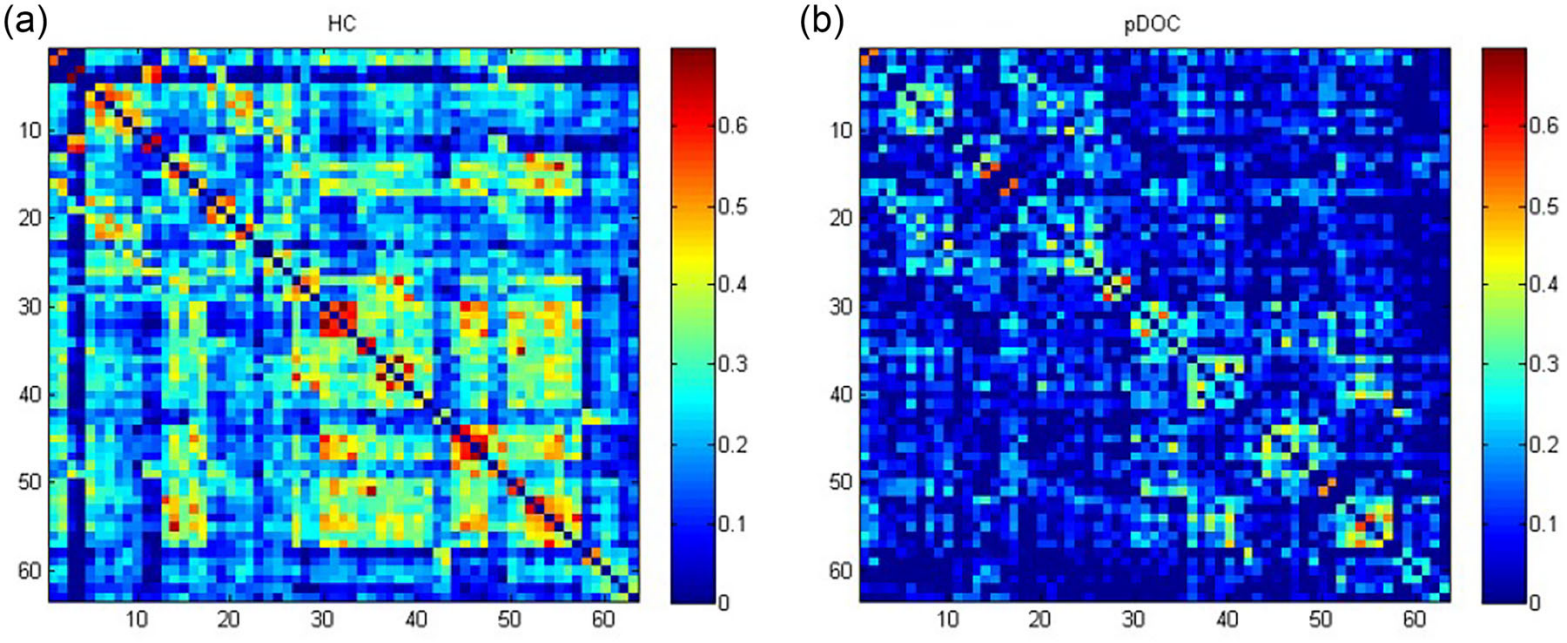
Whole‐brain mean functional connectivity strength based on deoxyhemoglobin (HbR) in the healthy adult group (a) and prolonged disorders of consciousness (pDOC) group (b).

**FIGURE 5:**
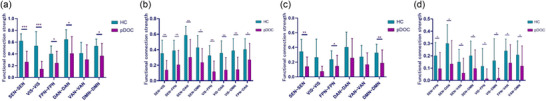
Statistical analysis of the differences in the strength of functional connectivity of each network in the cerebral cortex between the healthy adult group and the prolonged disorders of consciousness (pDOC) group. (a) Differential analysis of functional connectivity strength in homologous cortical brain networks based on oxyhemoglobin (HbO), (b) differential analysis of functional connectivity strength in heterologous cortical brain networks based on HbO, (c) differential analysis of functional connectivity strength in homologous cortical brain networks based on deoxyhemoglobin (HbR), and (d) differential analysis of functional connectivity strength in heterologous cortical brain networks based on HbR. HC, control group.

Based on HbR to calculate the whole‐brain functional connectivity of the two groups, the functional connectivity strength of the pDOC group in each brain network was still lower than that of the HC group (Figures 4). Analyses of the strength of functional connectivity of homologous brain networks showed that three brain networks were significantly reduced in the pDOC group (Figure 5c). Analyses of the strength of functional connectivity of heterologous brain networks showed that eight brain networks were significantly reduced in the pDOC group (Figure 5d).

### 3.4 Univariate binary logistic regression analysis

Brain networks with statistically significant differences were analyzed by univariate binary logistic regression, *B* is the parameter estimate, SD is the standard error, OR is the odds ratio, Wals is the Wald value, and 95% CI is the 95% confidence interval, and two‐tailed *p* < .05 was considered a statistically significant difference. From Table 2, it can be seen that the connection strengths of HbO‐based homologous brain functional connection networks SEN∼SEN, VIS∼VIS, DAN∼DAN, and DMN∼DMN were related factors reflecting the participants' consciousness. It can be seen from Table 3 that the connection strengths of HbO‐based heterogeneous brain functional connection networks SEN∼VIS, SEN∼FPN, SEN∼DAN, SEN∼DMN, VIS∼FPN, VIS∼DAN, and VIS∼DMN were related factors reflecting the participants' consciousness. The univariate binary logistic analyses of the connection strengths of the homologous and heterologous brain functional connectivity networks based on HbR are shown in Tables 4 and 5, respectively, and the connection strengths of SEN∼SEN as well as SEN∼DAN were related factors reflecting the participants' consciousness.

**TABLE 2:** Univariate binary logistic regression analysis of functional connectivity strength in homologous brain networks based on oxyhemoglobin (HbO).

Brain networks	*B*	SD	Wals	*p*	OR	95%CI
						Lower∼upper
SEN∼SEN	11.7642	4.4002	7.1478	*p =*.0075	128,565.7582	23.1018∼715,493,417.8459
VIS∼VIS	11.8869	5.1345	5.3597	*p =*.0206	145,343.3338	6.1935∼3,410,797,561.5800
FPN∼FPN	5.8680	3.0098	3.8010	*p =*.0512	353.5277	0.9693∼128,936.9457
DAN∼DAN	4.7125	2.2311	4.4612	*p =*.0347	111.3346	1.4044∼8826.3802
DMN∼DMN	6.4871	3.2363	4.0178	*p =.0450	656.6086	1.1548∼37,3326.7996

Abbreviations: *B*, parameter estimate; CI, confidence interval; DAN, dorsal attentional network; DMN, default mode network; FPN, frontoparietal network; OR, odds ratio; SEN, sensorimotor network; VAN, ventral attentional network; VIS, visual network.

*p < 0.05 and **p < 0.01.

**TABLE 3:** Univariate binary logistic regression analysis of functional connectivity strength in heterologous brain networks based on oxyhemoglobin (HbO).

Brain networks	*B*	SD	Wals	*P*	OR	95%CI
						Lower∼upper
SEN∼VIS	10.2354	4.3622	5.5056	*p =.0190	278,72.5307	5.3962∼143,968,771.9459
SEN∼FPN	7.4841	3.3822	4.8963	*p =.0269	1779.5234	2.3515∼1,346,689.1202
SEN∼DAN	8.2834	3.2695	6.4189	*p =.0113	3957.6364	6.5231∼2,401,154.1638
SEN∼DMN	6.7526	2.9876	5.1086	*p =.0238	856.3202	2.4523∼299,018.6027
VIS∼FPN	12.5561	5.0893	6.0867	*p =.0136	283,807.8424	13.2122∼6,096,412,188.1890
VIS∼DAN	8.5432	3.5859	5.6760	*p =.0172	5131.6828	4.5490∼5,788,958.1226
VIS∼DMN	9.3019	3.6447	6.5136	*p =.0107	10,958.7120	8.6578∼13,871,199.2020
FPN∼DAN	4.8792	2.8506	2.9298	*p =*.0870	131.5285	0.4927∼35,109.1642

Abbreviations: *B*, parameter estimate; CI, confidence interval; DAN, dorsal attentional network; DMN, default mode network; FPN, frontoparietal network; OR, odds ratio; SEN, sensorimotor network; VAN, ventral attentional network; VIS, visual network.

*p < 0.05 and **p < 0.01.

**TABLE 4:** Univariate binary logistic regression analysis of functional connectivity strength in homologous brain networks based on deoxyhemoglobin (HbR).

Brain networks	B	SD	Wals	*p*	OR	95%CI
						Lower∼upper
SEN∼SEN	10.7331	4.8957	4.8063	*p =.0284	45,847.0605	3.1194∼673,840,262.9886
FPN∼FPN	6.3142	4.2183	2.2406	*p =*.1344	552.3469	0.1418∼2,151,798.2742
DMN∼DMN	7.8377	4.0858	3.6798	*p =*.0551	2534.4388	0.8434∼7,616,053.4029

Abbreviations: *B*, parameter estimate; CI, confidence interval; DMN, default mode network; FPN, frontoparietal network; OR, odds ratio; SEN, sensorimotor network.

*p < 0.05 and **p < 0.01.

**TABLE 5:** Univariate binary logistic regression analysis of functional connectivity strength in heterologous brain networks based on deoxyhemoglobin (HbR).

Brain networks	B	SD	Wals	*p*	OR	95%CI
						Lower∼upper
SEN∼FPN	8.4209	4.6485	3.2816	*p =*.0701	4541.1250	0.5016∼41,113,648.8053
SEN∼DAN	6.2918	3.1899	3.8904	*p =.0486	540.1060	1.0405∼280,371.5232
SEN∼VAN	8.4876	4.9437	2.9476	*p =*.0860	4854.2810	0.3006∼78,380,525.0520
SEN∼DMN	6.8500	4.1226	2.7609	*p =*.0966	943.9066	0.2923∼3,048,459.0512
VIS∼FPN	10.0148	5.8300	2.9509	*p =*.0858	22,355.2301	0.2437∼2,050,424,945.8928
VIS∼DMN	12.6543	6.7533	3.5111	*p =*.0610	313,114.1673	0.5588∼175,446,010,870.6136
FPN∼VAN	7.5199	4.3529	2.9845	*p =*.0841	1844.3911	0.3636∼9,355,241.0966
VAN∼DMN	5.8191	4.0446	2.0670	*p =*.1502	336.6625	0.1215∼933,111.3983

Abbreviations: *B*, parameter estimate; CI, confidence interval; DAN, dorsal attentional network; DMN, default mode network; FPN, frontoparietal network; OR, odds ratio; SEN, sensorimotor network; VAN, ventral attentional network; VIS, visual network.

*p < 0.05 and **p < 0.01.

### 3.5 ROC curve analysis

To further explore the cut‐off value of each brain functional connectivity network reflecting the participant's state of consciousness, ROC curves of their relationship with the participant's state of consciousness were plotted in this study.

#### 3.5.1 HbO‐based brain network

**FIGURE 6:**
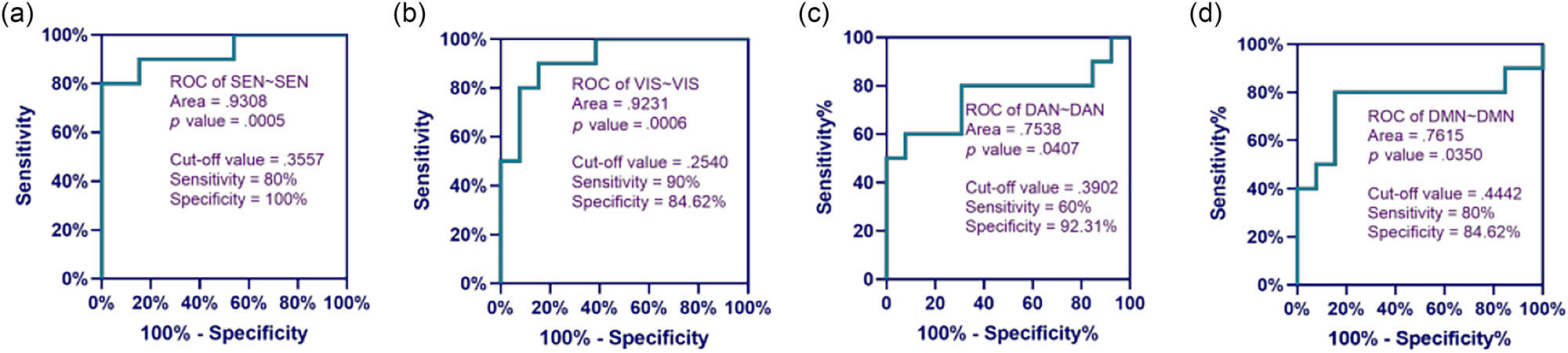
Receiver operating characteristic (ROC) curve analysis of functional connectivity strength of homologous cortical brain networks based on oxyhemoglobin (HbO). (a) ROC of SEN∼SEN, (b) ROC of VIS∼VIS, (c) ROC of DAN∼DAN, and (d) ROC of DMN∼DMN. DAN, dorsal attentional network; DMN, default mode network; SEN, sensorimotor network; VAN, ventral attentional network; VIS, visual network.

**FIGURE 7:**
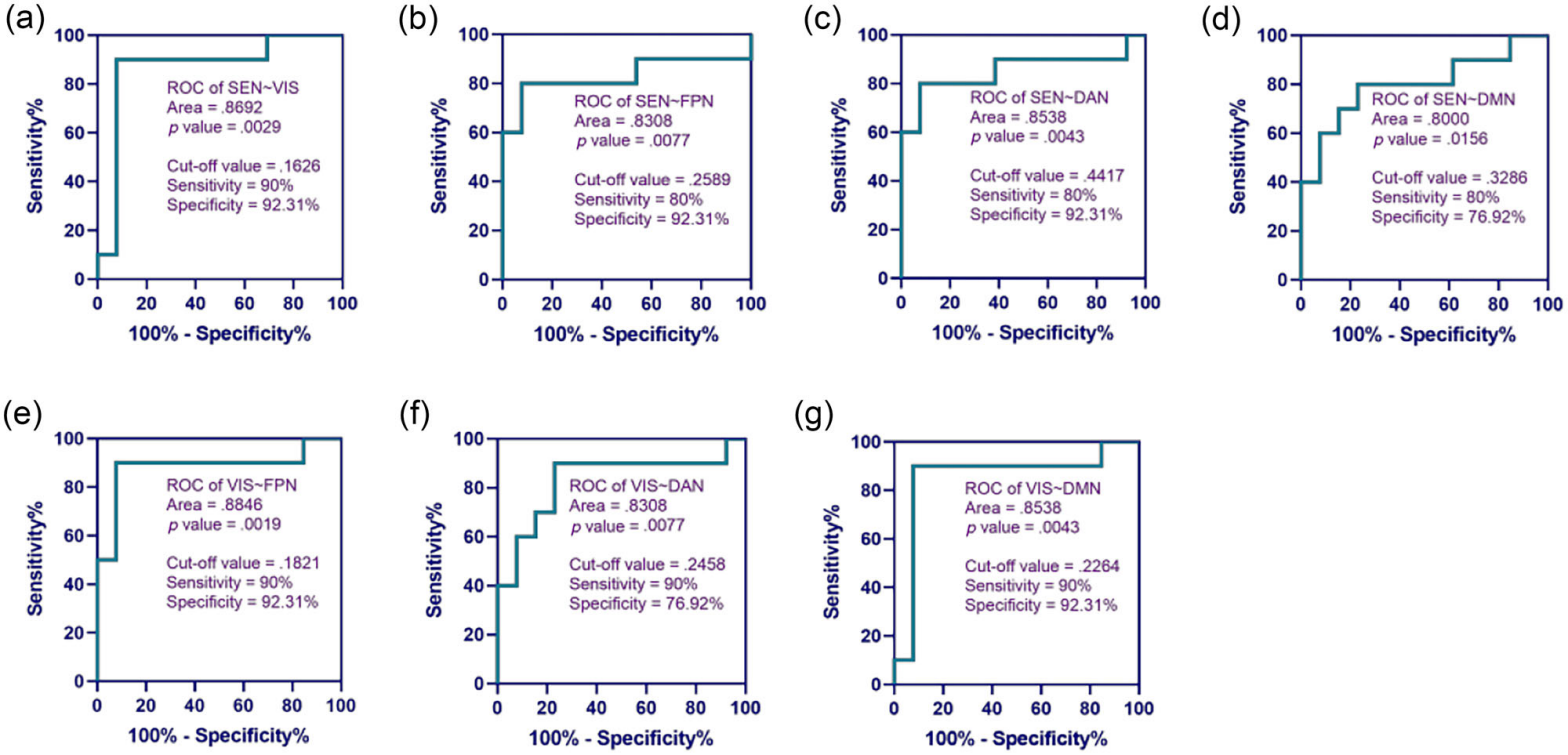
Receiver operating characteristic (ROC) curve analysis of functional connectivity strength of heterogeneous cortical brain networks based on oxyhemoglobin (HbO). (a) ROC of SEN∼VIS, (b) ROC of SEN∼FPN, (c) ROC of SEN∼DAN, (d) ROC of SEN∼DMN, (e) ROC of VIS∼FPN, (f) ROC of VIS∼DAN, and (g) ROC of VIS∼DMN. DAN, dorsal attentional network; DMN, default mode network; FPN, frontoparietal network; SEN, sensorimotor network; VAN, ventral attentional network; VIS, visual network.

As can be seen from Figure 6, when the resting‐state functional connectivity strengths of the HbO‐based homologous brain functional connectivity networks SEN∼SEN, VIS∼VIS, DAN∼DAN, and DMN∼DMN were greater than or equal to 0.3557, 0.2540, 0.3902, and 0.4442, respectively, the probability of participants awakening was higher. The areas under the curves for SEN∼SEN and VIS∼VIS were the largest, 0.9308 and 0.9231, respectively, which means that SEN∼SEN and VIS∼VIS were more capable of predicting the participants' state of consciousness. Figure 7 shows that when the resting functional connectivity strengths of HbO‐based heterogeneous brain functional connection networks SEN∼VIS, SEN∼FPN, SEN∼DAN, SEN∼DMN, VIS∼FPN, VIS∼DAN, and VIS∼DMN were greater than or equal to 0.1626, 0.2589, 0.4417, 0.3286, 0.1821, 0.2458, and 0.2264, respectively, the probability of participants awakening was greater, with SEN∼VIS and VIS∼FPN predicting participant's state of consciousness more strongly.

#### 3.5.2 HbR‐based brain network

**FIGURE 8:**
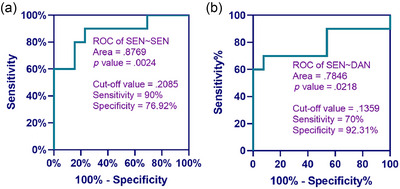
Receiver operating characteristic (ROC) curve analysis of functional connectivity strength of cortical brain networks based on HbR. (a) ROC of SEN∼SEN and (b) ROC of SEN∼DAN. SEN, sensorimotor network; DAN, dorsal attentional network.

ROC curve analysis of homologous and heterologous brain functional connection networks based on HbR is shown in Figure 8a and b, respectively. When the resting‐state functional connectivity strengths of SEN∼SEN and SEN∼DAN were greater than or equal to 0.2085 and 0.1359, respectively, the probability of participants' awakening was higher. The area under the curve for SEN∼SEN is the largest at 0.8769, which means that SEN∼SEN has a higher ability to predict the participant's state of consciousness.

## 4 DISCUSSION

In this study, we explored the characteristics and differences in resting‐state functional connectivity of cortical brain networks in pDOC and healthy adults by fNIRS and found that the strength of functional connectivity was lower in the pDOC group than in the healthy adult group, and that this trend was present in both homologous and heterologous brain networks. Relevant factors reflecting the participant's state of consciousness were also explored, providing a preclinical marker for predicting arousal in patients with pDOC.

### 4.1 Sensitivity and specificity of detecting the strength of functional connectivity in cortical brain networks based on HbO signal and HbR signal

fNIRS can detect changes in the concentration of HbO and HbR in the cerebral cortex of subjects in real time. Theoretically, HbO and HbR are highly correlated. However, in practical use, there is noise interference in real HbO and HbR data, and recording HbO and HbR at the same time can significantly increase the correct rate of brain‐computer interface (Cui et al., 2010). So, we used both HbO and HbR signals from patients. The results were as follows: first, compared to the healthy adult group, the whole‐brain mean functional connectivity strength either based on HbO or HbR was significantly decreased in the pDOC group. Second, the results of intergroup comparison of HbO‐based homologous brain networks showed a significant decrease in the functional connectivity strength of five brain networks in the pDOC group, which included the results of intergroup comparison of HbR‐based homologous brain networks, and the results of intergroup comparison of both HbO‐ and HbR‐based heterologous brain networks showed a significant decrease in the functional connectivity strength of eight brain networks, of which five heterologous brain networks were overlapped. Finally, univariate binary logistic regression analysis and ROC curve analysis showed that 11 of the HbO‐based brain networks could reflect the subjects' conscious state, while only two of the HbR‐based brain networks could reflect the subjects' conscious state and were included in the 11 HbO‐based brain networks. The above results show that the sensitivity of the study using the HbO signals is higher than that using HbR signals, and the specificity of the study using HbR signals is higher than that using HbO signals.

### 4.2 Decreased strength of functional connectivity in pDOC cortical brain networks

Consciousness consists of two dimensions: the state of arousal and the content of consciousness. The cerebral cortical system, the thalamic system, and the brainstem ascending reticular activation system play an important role in maintaining human consciousness (Jang et al., 2019; Schiff, 2010). pDOC stems from direct interference with the neural systems that regulate arousal and consciousness, as well as indirect interference with the connections between these systems. The central loop model suggests that the direct deafferentation effects of central thalamic neurons and the cumulative effects of inhibition of striatal intermediate‐type multispinal neurons (MSNs) activity combine to result in widespread reductions in synaptic activity throughout the brain and reduced brain metabolic rates, ultimately producing a range of unresponsive symptoms in patients with pDOC (Zheng et al., 2023). At the same time, recovery of consciousness is thought to be closely related to the restoration of connectivity within cortical thalamic neuronal activity (Edlow et al., 2021). Therefore, the present study used fNIRS to directly detect the strength of functional network connectivity in the brains of pDOC patients by bypassing behavioral responses, which could reveal the hidden consciousness and cognition in the brains of some patients earlier, thus providing a means for more accurate diagnosis and more accurate prognosis. In this study, we found that the strength of functional connectivity of homologous and heterologous brain networks in the pDOC group was lower than that in the healthy adult group. This suggests that pDOC diminishes the synergy between brain regions, and that by looking at the strength of functional connectivity, it may be possible to predict arousal in pDOC patients.

### 4.3 Brain networks reflecting subjects' states of consciousness

In this study, we captured six brain networks in the cerebral cortex, including SEN, DAN, VAN, DMN, FPN, and VIS. The SEN consists of the sensorimotor cortex, the supplementary motor area, and the secondary sensorimotor cortex, which are low‐level functional brain regions that are mainly responsible for sensory and motor‐related functions (Caspers et al., 2021). SEN is highly stable and is the first resting‐state network to be discovered (Biswal et al., 1995). Sensory transport cortical integration is impaired in patients with pDOC, and reduced levels of neuronal metabolism are associated with pDOC‐related hypomotor function. In the present study, we found that based on the functional connectivity strength calculated from HbO and HbR, the connectivity strength between FPN, DAN, DMN, and SEN in the pDOC group was significantly reduced, as well as the connectivity strength between the homologous brain networks of the SEN, which may indicate that the reduction of sensory‐motor functions as well as the impaired motor‐sensory conduction are the important influencing factors of pDOC. Moreover, ROC curve analysis revealed that the cut‐off value of SEN∼SEN brain functional connectivity strength, whether calculated on the basis of HbO or HbR, had the highest ability to predict the participant's state of consciousness. This shows that sensorimotor function is of great importance in the maintenance of the state of consciousness.

The DMN is an important resting‐state brain network, mainly involved in episodic memory, self‐reference, maintenance of arousal, and monitoring of the surrounding environment and is a high‐level cognitive network region (Greicius et al., 2003). Damage to the DMN is closely related to a variety of neurological and psychiatric disorders such as Alzheimer's disease (Y. Chen et al., 2021), Parkinson's disease (Ruppert et al., 2021), epilepsy (Gonen et al., 2020), and autism (M. Wang et al., 2020). By comparing resting‐state functional connectivity of different networks in MCS and UWS patients, Qin et al.(2015)found lower connectivity within the DMN in the UWS group, suggesting that the DMN predicts recovery of consciousness. In this study, the connection strength between VIS, SEN, and DMN in pDOC group were significantly reduced, and the connection strength between DMN homologous brain networks was also significantly reduced. When the functional connection strength of DMN∼DMN, SEN∼DMN, and VIS∼DMN calculated based on HbO were greater than or equal to 0.4442, 0.3286, and 0.2264, respectively, the probability of awakening was greater, which suggests that the impaired ability to perceive the external environment is one of the key factors affecting arousal in patients with pDOC.

Attention represents the initial aspect of cognitive processing and is a selective activity of consciousness. The DAN is bilaterally lateralized, provides top‐down attentional orienting, and has an antagonistic relationship with the DMN (Mallas et al., 2021). In the present study, we found that the DAN was the network with the highest strength of functional connectivity among the six brain networks. And based on the functional connectivity strength calculated from HbO and HbR, the connectivity strength between SEN and DAN in the pDOC group was significantly reduced. The probability of participants awakening was greater when the resting‐state functional connectivity strength of SEN∼DAN calculated based on HbO was greater than or equal to 0.4417 and when the resting‐state functional connectivity strength of SEN∼DAN calculated based on HbR was greater than or equal to 0.1359. This implies that decreased connectivity between sensorimotor and attentional areas in patients with pDOC may affect their arousal.

The VAN has a right lateralization, is primarily responsible for non‐spatial attention, and can be involved in stimulus‐driven top‐down attentional selection (Vossel et al., 2014). Deslauriers et al. (2017) found in fMRI that functional connectivity of brain regions in the posterior part of the VAN is elevated and that functional connectivity of brain regions in the anterior part of the VAN is reduced in older adults. In this study, the connection strength between homologous and heterologous brain networks of the VAN in the pDOC group did not differ from that of the healthy adult group based on the functional connection strength calculation of HbO; functional connectivity strength calculations based on HbR showed that the strength of heterologous brain network connections between SEN, FPN, DMN, and VAN were lower than in the healthy adult group. This result may be related to the inconsistent trend of functional connectivity changes in the anterior and posterior parts of the VAN. Therefore, this study does not recommend the functional connectivity strength of the VAN as a predictor of pDOC arousal.

FPN is mainly responsible for higher cognitive activities including processing and regulation of information processing of language, attention, vision, memory, and other related cognitive processes (Seeley et al., 2007). Long et al. (2016) showed that interactions between the dorsolateral prefrontal cortex in the FPN and the precuneus within the DMN may play a role in regulating consciousness, with greater connectivity between the two suggesting a greater likelihood of recovery of consciousness. In the present study, the connection strength between SEN, VIS, VAN, and FPN was significantly reduced in the pDOC group, as well as between FPN homologous brain networks. The connection strength between DMN and FPN was also reduced, but the difference was not significant, which may be related to the small sample size of this study. This may demonstrate that dysfunction of higher cognitive activities and its connection impairment with sensorimotor, visual, and memory is one of the main mechanisms of pDOC. In addition, the ROC curve analysis shows that the area under the curve of VIS∼FPN brain functional connection strength calculated based on HbO is 0.8846, which ranks third in predicting the conscious state of the subjects in all brain functional connection networks and can be used as the main preclinical biomarker for predicting the awakening state of pDOC patients.

The occipital region is the brain area where the VIS is located, with extensive fiber connections to the frontal and temporal lobes, and belongs to the lower brain functional areas (Yang et al., 2017). In this study, the connection strengths between FPN, DMN, and VIS were significantly reduced in the pDOC group. This suggests that visual dysfunction affects arousal in patients with pDOC. However, due to the difficulty of fNIRS in acquiring signals in the occipital region, we only lined up four channels, which did not fully cover the VIS‐related region, and this result is subject to further refinement.

## 5 LIMITATION

First, the small sample size included in this study did not allow for a stratified analysis of the etiology and severity of the disease in pDOC patients. Second, fNIRS is an emerging noninvasive functional brain imaging technique in recent years, and its greatest advantages are its faster temporal resolution than fMRI techniques, its higher spatial resolution than EEG and SSEP techniques, and, more importantly, its portability and low artifact interference. Although its agreement with fMRI findings is high, there is a lack of comparison of its findings with those of EEG and SSEP, and electrophysiological indices such as EEG and SSEP should be tested simultaneously in this study to validate the results of the trial. In future studies, our group will try to expand the sample size and combine neuroimaging tools such as electrophysiology and fMRI to delve deeper into the brain networks associated with pDOC, and to further clarify the markers that predict pDOC arousal. Finally, this study covered six cortical brain networks with 63 channels, and the VIS was incompletely covered with only four channels. In future studies, we will choose the fNIRS headcap, which covers a wider range of brain regions, to explore pDOC‐related mechanisms in depth, and to promote precise rehabilitation and treatment of pDOC.

## 6 CONCLUSION

In conclusion, pDOC is associated with extensive brain network changes affecting its short‐ and long‐range connections, suggesting a possible mechanism for impaired consciousness in pDOC patients. Although additional experiments are necessary, cut‐off values of brain networks reflecting participants' state of consciousness could serve as potential preclinical markers for predicting arousal in pDOC patients.

## AUTHOR CONTRIBUTIONS

**Yaomin Luo**: Data curation; writing—original draft; visualization; software; validation. **Lingling Wang**: Conceptualization; methodology; data curation; investigation; formal analysis; validation. **Yuxuan Yang**: Conceptualization; methodology; investigation; formal analysis. **Xin Jiang**: Data curation; supervision; resources; software. **Kaiyuan Zheng**: Software; resources; supervision; data curation. **Yu Xi**: Investigation; validation. **Min Wang**: Investigation; conceptualization. **Li Wang**: Conceptualization; investigation; funding acquisition. **Yanlin Xu**: Investigation; conceptualization. **Jun Li**: Conceptualization; investigation. **Yulei Xie**: Methodology; supervision; resources; project administration; formal analysis; writing—review and editing. **Yinxu Wang**: Methodology; data curation; supervision; resources.

## CONFLICT OF INTEREST STATEMENT

The authors declare no conflicts of interest.

### PEER REVIEW

The peer review history for this article is available at https://publons.com/publon/10.1002/brb3.70002.

## Data Availability

Anonymized data will be available on request.
